# Supplementary figures and images for: Indicators to assess temporal variability in marine connectivity processes: A semi-theoretical approach
Source: PLoS One. 2024 Jul 1;19(7):e0297730. doi: 10.1371/journal.pone.0297730 (PMC11216624; doi:10.1371/journal.pone.0297730)

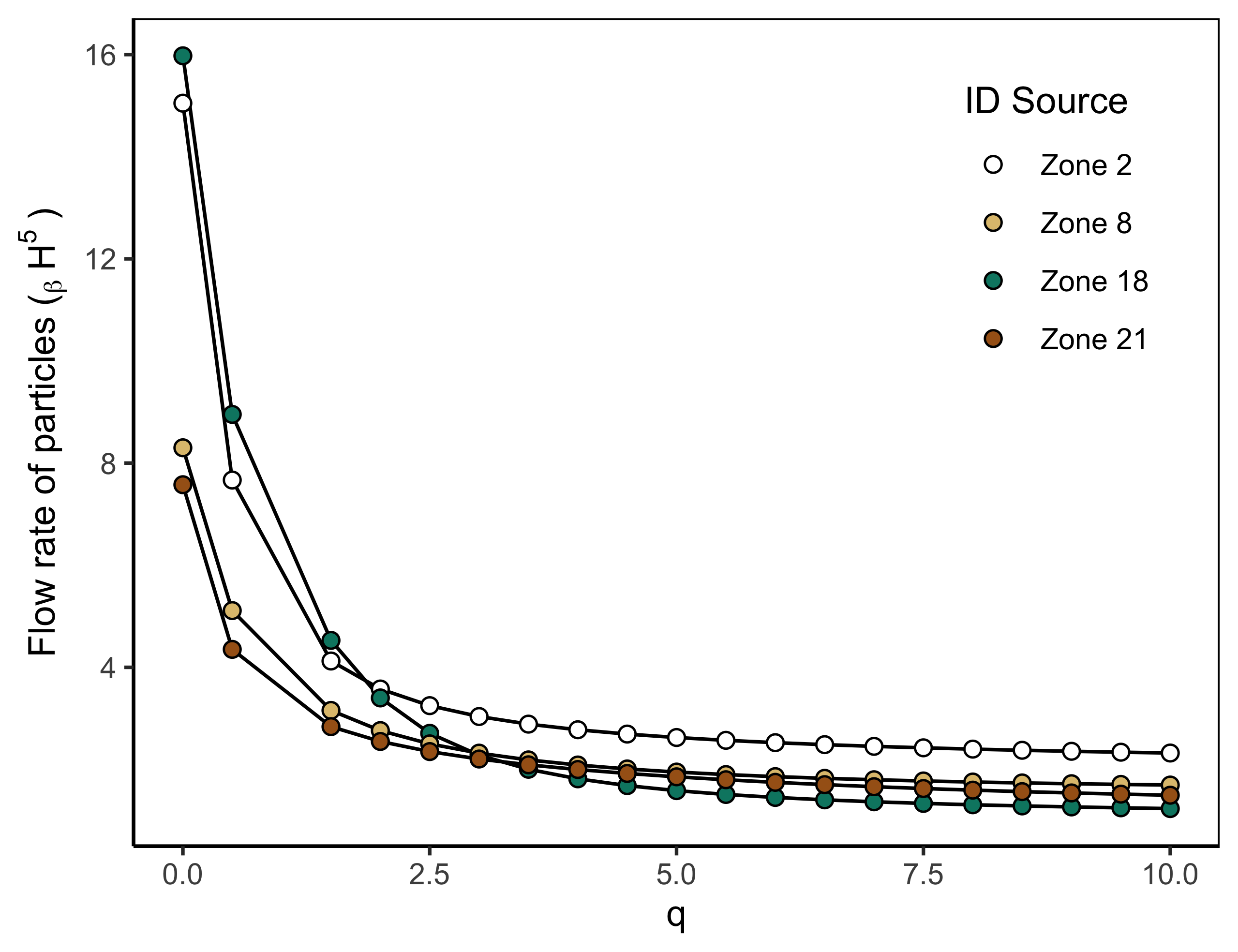

Supplement: S1 Fig — Following the elbow approach, we selected q = 5. (TIFF) [file pone.0297730.s001.tiff]

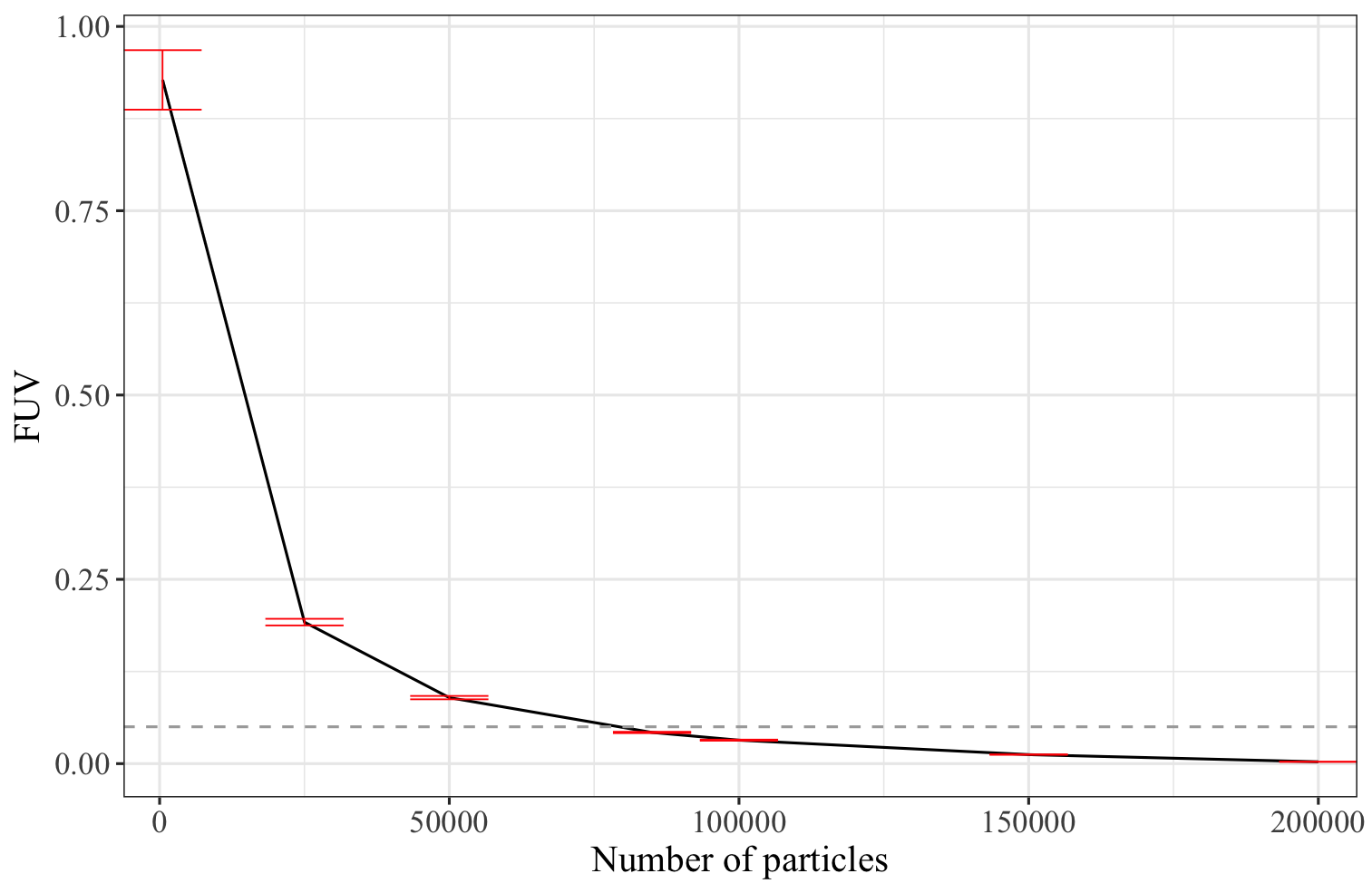

Supplement: S2 Fig — The dashed grey lines indicate the threshold of 0.05, below which, the number of particles represents 95% of the particle dispersal variability. The threshold is reached for 85,000 particles. Red error bars show the standard deviation of FUV around the mean. (TIF) [file pone.0297730.s002.tif]

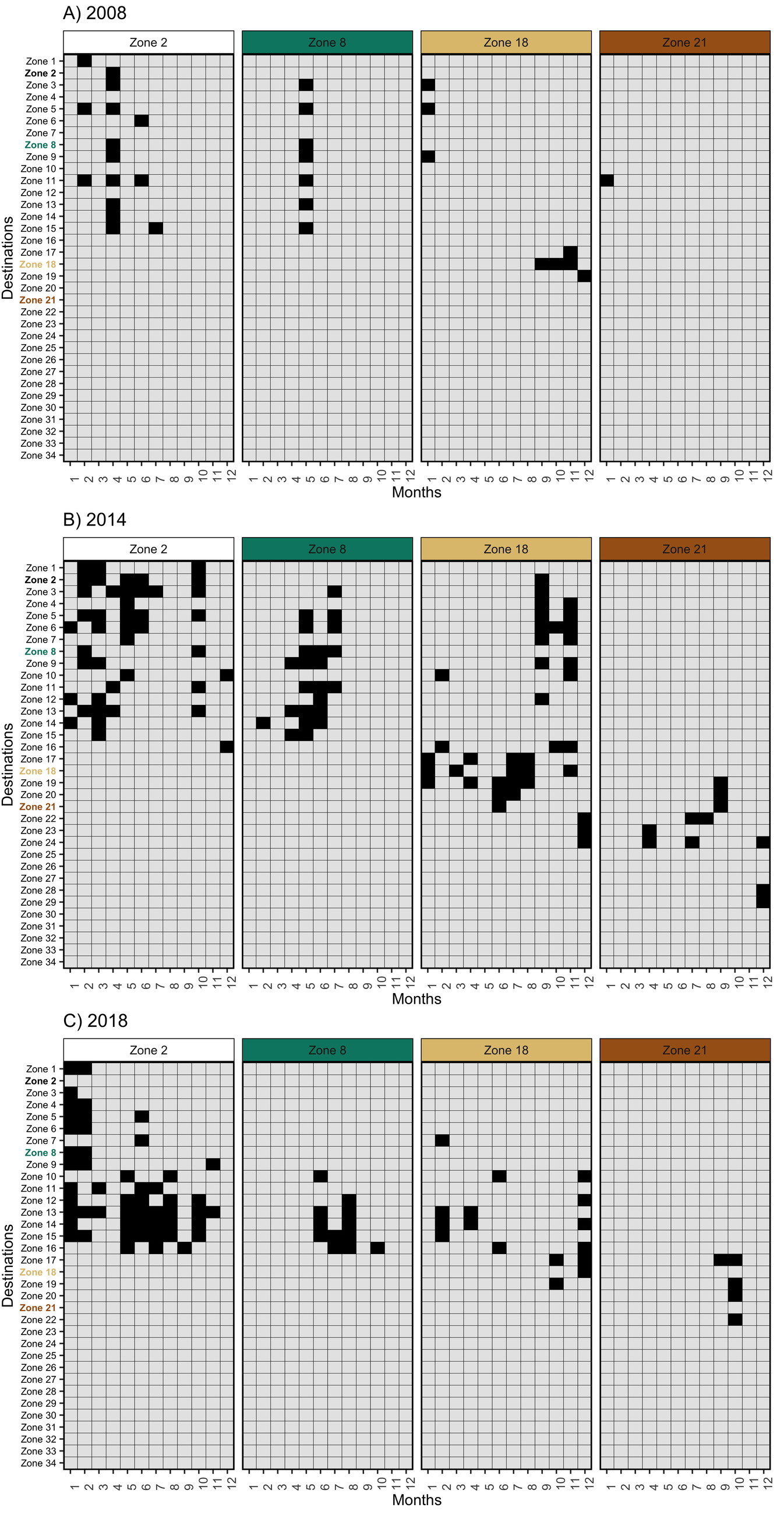

Supplement: S3 Fig — Monthly connectivity occurrence (X-axis) from a source zone to a destination zone for the years A) 2008, B) 2014, and C) 2018. (TIF) [file pone.0297730.s003.tif]

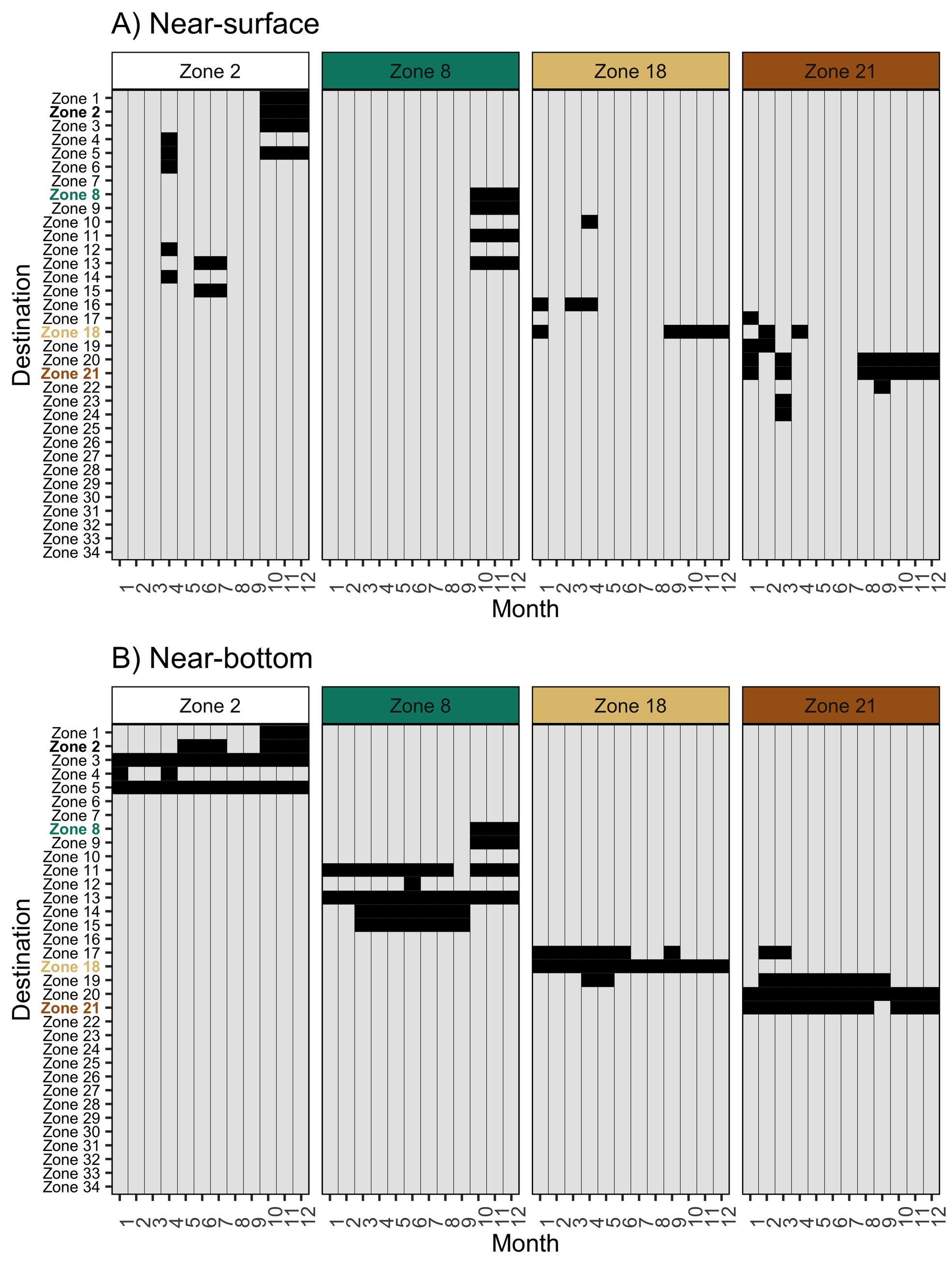

Supplement: S4 Fig — Occurrence and flow of connectivity over months from a source zone (facets) to a destination zone for the particle release A) near surface and B) near bottom. (TIF) [file pone.0297730.s004.tif]
