# Supplementary material for: Indicators to assess temporal variability in marine connectivity processes: A semi-theoretical approach
Source: PLoS One. 2024 Jul 1;19(7):e0297730. doi: 10.1371/journal.pone.0297730 (PMC11216624; doi:10.1371/journal.pone.0297730)
Supplement: S1 File — (DOCX) [file pone.0297730.s005.docx]

**S1 Appendix**

**Theoretical example for calculating Connectivity indicators and comparison with Graph Network theory indicators**

*Open Research statement: A R code (doi:10.6084/m9.figshare.24680793) is publicly archived in the Figshare repository to support this theoretical example.*

The example considered the connectivity from the source ‘Zone A’ during d=3 continuous years. Every year, N_t_=70 particles were connected to up to f=7 destinations zones named “A to G”. Over the time d, N=210 particles were connecting MPA A to itself and the six other MPAs. Here, T=d, as connections occurred during the three years. The resulting distribution of particles from Zone A to destinations are represented in Table A1.

Table A1. Theoretical dispersion of particles from Zone A to seven zones (A-G) for three consecutive years.

|  | **Year 1** | **Year 2** | **Year 3** |
| --- | --- | --- | --- |
| Zone A | 0 | 50 | 10 |
| Zone B | 0 | 0 | 10 |
| Zone C | 0 | 0 | 10 |
| Zone D | 10 | 0 | 10 |
| Zone E | 10 | 10 | 10 |
| Zone F | 0 | 0 | 10 |
| Zone G | 50 | 10 | 10 |

The information from X_ij_ (the quantity of linked sites *j* over time), S_Xij_ (a binary index of repeated links) x_ij_ (a vector of values representing the durations of uninterrupted connections to a given linked site *j*), max(x_ij_), and N_ij_ (the link weight between a focus site i and linked sites *j* across time) are provided in Table A2 to help with the calculation of the indicator.

Table A2. Values of five variables used to calculate connectivity indicators for Zone A.

| **X_ij_** | $\boldsymbol{S}_{\boldsymbol{X}_{\boldsymbol{ij}}}$ | **x_j_** | **max(x_j_)** | **N_j_** |
| --- | --- | --- | --- | --- |
| X_A_ = 2 | $S_{X_{A}}=1$ | x_A_ = [2] | max(x_A_) = 2 | N_A_ = 60 |
| X_B_ = 1 | $S_{X_{B}}=0$ | x_B_ = [1] | max(x_B_) = 1 | N_B_ = 10 |
| X_C_ = 1 | $S_{X_{C}}=0$ | x_C_ = [1] | max(x_C_) = 1 | N_C_ = 10 |
| X_D_ = 2 | $S_{X_{D}}=1$ | x_D_ = [1, 1] | max(x_D_) = 1 | N_D_ = 20 |
| X_E_ = 3 | $S_{X_{E}}=1$ | x_E_ = [3] | max(x_E_) = 3 | N_E_ = 30 |
| X_F_ = 1 | $S_{X_{F}}=0$ | x_F_ = [1] | max(x_F_) = 1 | N_F_ = 10 |
| X_G_ = 3 | $S_{X_{G}}=1$ | x_G_ = [3] | max(x_G_) = 3 | N_G_ = 70 |

Occurrence indicators are:

$P_{X>1}= \frac{1*2+0+0+1*2+1*3+0+1*3}{2+1+1+2+3+1+3}= \frac{10}{13}=0.77$, $n_{link}=\frac{2+1+1+2+3+1+3}{T}=\frac{13}{3}=4.33$

Flux indicators are:

${}_{\alpha}{H^{5}}= \left[ \frac{70}{210} .\left\{ \left. 2*\left( \frac{10}{70} \right. \right)^{5}+ \left( \left. \frac{50}{70} \right) \right.^{5} \right\}+ \frac{70}{210} .\left\{ \left. \left( \frac{50}{70} \right. \right)^{5}+ 2* \left( \frac{10}{70} \right)^{5} \right\}+\frac{70}{210} .\left\{ 7*\left. \left( \frac{10}{70} \right. \right)^{5} \right\} \right]^{\frac{1}{-4}}$ = 1.68

${}_{\gamma}{H^{5}}=\left[ 3*\left( \frac{10}{210} \right)^{5}+ \left( \frac{20}{210} \right)^{5}+\left( \frac{30}{210} \right)^{5}+{\left. \left( \frac{60}{210} \right. \right)^{5}+\left( \frac{70}{210} \right)}^{5} \right]^{\frac{1}{-4}}$ = 3.58

${}_{\beta}{H^{5}}= \frac{3.58}{1.68}=2.12$, $P_{N|X=1}= \frac{30}{210}=0.14$

Frequency indicators are:

$F=$ $\frac{2+1+1+2+3+1+3}{7} .\frac{1}{3}=0.62$, $M_{link}=\frac{12}{7}. \frac{1}{3}=0.57$

**Comparison with Graph-theoretic approach.**

We calculated the values of three indicators from the Graph-Theoretic approach (Table A3): out-strength and degree based on the connectivity example provided in Table A1 using the average of the three years or the dataset across time. Out-strength and out-degree are the measures that fall close to the indicators, N_i,t_ and X_ij_, respectively.

Table A3. Two indicators of the graph-theoretic approach applied on the theoretical example

given in Table 1.

| Indicators | On average over time | On the whole dataset |
| --- | --- | --- |
| Weighted Out-strength | 70 | 210 |
| Out-Degree | 7 | 13 |
